# Wheat/Gluten-Related Disorders and Gluten-Free Diet Misconceptions: A Review

**DOI:** 10.3390/foods10081765

**Published:** 2021-07-30

**Authors:** Carolina Sabença, Miguel Ribeiro, Telma de Sousa, Patrícia Poeta, Ana Sofia Bagulho, Gilberto Igrejas

**Affiliations:** 1 Department of Genetics and Biotechnology, University of Trás-os-Montes and Alto Douro, 5000-801 Vila Real, Portugal; carolinasabenca@hotmail.com (C.S.); jmribeiro@utad.pt (M.R.); telmaslsousa@hotmail.com (T.d.S.); 2 Functional Genomics and Proteomics Unity, University of Trás-os-Montes and Alto Douro, 5000-801 Vila Real, Portugal; 3 LAQV-REQUIMTE, Faculty of Science and Technology, University Nova of Lisbon, 2829-546 Lisbon, Portugal; ppoeta@utad.pt; 4 Microbiology and Antibiotic Resistance Team (MicroART), Department of Veterinary Sciences, University of Trás-os-Montes and Alto Douro, 5000-801 Vila Real, Portugal; 5 National Institute for Agrarian and Veterinarian Research (INIAV), Estrada Gil Vaz, Ap. 6, 7350-901 Elvas, Portugal; ana.bagulho@iniav.pt

**Keywords:** wheat, nutrients, celiac disease, wheat allergy, non-celiac wheat/gluten sensitivity

## Abstract

In the last 10,000 years, wheat has become one of the most important cereals in the human diet and today, it is widely consumed in many processed food products. Mostly considered a source of energy, wheat also contains other essential nutrients, including fiber, proteins, and minor components, such as phytochemicals, vitamins, lipids, and minerals, that together promote a healthy diet. Apart from its nutritional properties, wheat has a set of proteins, the gluten, which confer key technical properties, but also trigger severe immune-mediated diseases, such as celiac disease. We are currently witnessing a rise in the number of people adhering to gluten-free diets unwarranted by any medical need. In this dynamic context, this review aims to critically discuss the nutritional components of wheat, highlighting both the health benefits and wheat/gluten-related disorders, in order to address common misconceptions associated with wheat consumption.

## 1. Introduction

The domestication of wheat revolutionized the human diet as this cereal provided a significant source of energy. Globally, wheat accounts for the largest harvested area of any crop [1] and provides more protein and calories than any other cereal crop [2]. Wheat is nutritious, simple to transport and store, and can be transformed into several types of food. The most valuable modern wheat species are hexaploid bread wheat (*Triticum aestivum* L.) and tetraploid durum wheat (*T. turgidum* L. var. *durum*), which have distinct genomes, grain composition, and end-use quality attributes. Wheat adapts to all climatic conditions common in agricultural fields (except for the hot tropics), so globally, it is harvested all year round [3].

Wheat is a valuable source of essential nutrients, providing carbohydrate-based energy and fiber, protein, B vitamins, calcium, magnesium, phosphorus, potassium, zinc, and iron [4]. In low and medium-income countries, grain-based foods still make up the central part of the diet. The wheat seed can be ground into flour or semolina, for example, which form the essential ingredients of bread, pasta, noodles, and other food products, essentially the primary source of nutrients for most of the world population [5]. Conversely, the lack of grains too often signifies hunger and malnutrition. The characteristic that has given wheat an advantage over other temperate crops is the unique viscoelastic properties of dough formed from wheat flours, which allow it to be processed into such an array of forms [6]. Dough viscoelasticity depends on the structures and interactions that occur between grain storage proteins that form the gluten protein complex [7].

Gluten, which is now an almost ubiquitous ingredient in the food industry, is implicated in several immune-mediated disorders, such as celiac disease (CD). Both CD and other intolerances are of increasing concern [8,9], and the prevalence of CD is predicted to rise [10]. These disorders demand a gluten-free diet (GFD), but a GFD can itself be associated with digestive problems due to insufficient intake of dietary fiber and other nutrients [11].

This review focuses on wheat from a human health perspective. We will present the positive impacts of wheat, referring to the benefits of the different components of the wheat grain on human health, and juxtapose this with the negative impacts on the health of sensitive and genetically susceptible individuals caused by wheat components. At the same time, we draw attention to common gluten-related misconceptions and try to demystify them.

## 2. The Health Benefits of Wheat

**Figure 1:**
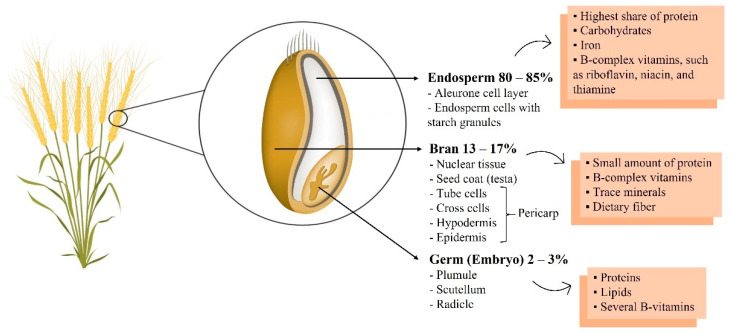
Wheat grain constitution.

Wheat grain is composed of the germ (2–3%), the bran (13–17%), and the endosperm (80–85%) [5] (Figure 1). Wheat germ is the embryo of the wheat kernel and is relatively rich in protein, lipids, and several of the B-vitamins [5,12]. Whole-wheat flour includes the bran, which contains a limited amount of protein, larger quantities of the B-complex vitamins, trace minerals, and indigestible cellulose material called dietary fiber [5,12]. White flour originates from the endosperm. The endosperm contains most of the protein in the whole kernel, iron, carbohydrates, and many B-complex vitamins, such as riboflavin, thiamine, and niacin [5,12].

The consumption of wheat brings many health benefits. In the European Prospective Investigation into Cancer and Nutrition (EPIC) study populations, 27% of total carbohydrate intake was from bread [13]. Epidemiological studies show that cereal dietary fiber and wholegrain consumption protects against the fast increasing chronic diseases related to a sedentary lifestyle, such as type 2 diabetes and cardiovascular disease [14,15,16,17].

### 2.1. Proteins

Protein is an essential nutrient for humans and animals [5]. Protein content is used to classify wheat. Breeders target this feature by regularly selecting for protein content traits in breeding programs; wheat with a low protein content is suitable for animal feed other uses, while wheat with a high protein content is necessary for breadmaking [4,7]. Protein content differs depending on the growing conditions, type or class of wheat, and fertilizer inputs, especially nitrogen [4,18]. Thus, there is no such thing as typical protein content, but on average, it can vary between 9–18% of the grain weight [4,5,19,20,21,22]. Protein is unequally distributed in the grain. A percentage of 5.1% of protein was reported in the pericarp, 5.7% in the testa, 22.8% in the aleurone, and 34.1% in the germ [21]. T.B. Osborne demonstrated that wheat proteins could be classified according to their extractability and solubility in distinct solvents [23]. Globulins are insoluble in pure water, and high NaCl concentrations but soluble in dilute NaCl solutions; albumins are soluble in water; glutenins are soluble in dilute acid or sodium hydroxide solutions, and gliadins are soluble in 70% ethyl alcohol [5].

Of the 20 amino acids commonly present in proteins, lysine, leucine, isoleucine, phenylalanine, threonine, tyrosine, tryptophan, histidine, valine, and methionine (and potentially cysteine since it can only be synthesized from methionine) are considered essential because they must be provided in the diet as animals cannot synthesize them [7]. Average contents of essential amino acids reported for whole wheat, wholemeal, and white flour are compared with the physiological requirements for older children, adolescents, and adults in Table 1. All cereals have a low content of lysine. In wheat, barley, rye, corn, and oats, methionine content is also low. Both amino acids are substantially lower in flour than in meat, milk, or egg proteins [24]. The data in Table 1 support the notion that lysine is the most limiting amino acid in wheat grains, with other essential amino acids being present in adequate amounts for older children, adolescents, and adults, making this cereal an excellent food for nutrition for any age group. Wheat breeders have attempted to improve the grain’s essential amino acid content. The approach has been effective in producing high-lysine barley and numerous maize cultivars [25]. Additional efforts were made using genetic engineering approaches to increase the synthesis and reduce the catabolism of these essential amino acids and express enriched recombinant proteins [26].

### 2.2. Carbohydrates

The wheat grain consists of 85% carbohydrate at maturity, 80% of which is the starchy endosperm. The non-starch carbohydrate is constituted of approximately 7% mono-, di-, and oligosaccharides and fructans, along with about 12% of cell wall polysaccharides [28,29]. In addition to being an essential energy source in the human nutrition and animal feed [30], wheat starch is the substrate for the production of alcoholic beverages and fuel ethanol by fermentation [31] and is the raw material for several other industries [32]. Polysaccharides are the main structural elements of the protoplasts walls present in all cells of the grain tissues. The cell wall polysaccharides are essential in human diet as sources of dietary fiber and have an impact on end-use quality and grain consumption [32].

Carbohydrates are recognized by WHO/FAO [33] as the macronutrient humans need to consume the most. Many countries have nutritional guidelines that emphasize the importance of cereals and cereal carbohydrates as the foundation of a healthy diet [34], mainly because the primary benefit of carbohydrates is as a source fuel, glucose. All body tissues, including brain tissue, require glucose. While the brain consist of only 2% of body mass, it uses 20% of the fuel [35]. Dietary carbohydrate is also vital in ensuring gastrointestinal integrity and function and glycemic homeostasis. Unlike protein and fat, high levels of complex carbohydrate are not associated with adverse health consequences to the extent that diets high in complex carbohydrates are less likely to lead to obesity and its morbid consequences than diets high in fat [36]. In an ideal diet, at least 55% of total energy should come from carbohydrates obtained from various food sources [37].

### 2.3. Lipids

Lipids are a minor constituent of wheat, mostly in the germ, making up 3–4% of the whole grain weight and 1–2.5% of directly milled flour [29]. Lipids have a critical role in baking processes, dough mixing, and the acceptance of the finished products by consumers. Their ability to associate with gluten proteins and form complexes contributes to stabilizing the gas-cell structure, significantly influencing the final texture of baked products and loaf volume [38].

Wheat grain lipids can be classified into polar and non-polar lipids. In all membranes we can find polar lipids such as phospholipids and glycolipids. Half of the total non-polar lipids in wheat are triglycerides that are deposited in spherosomes surrounded by monolayer membranes. The remaining non-polar lipids are mono- and diglycerides, sterol esters, and fatty acids. Wheat lipids can also be divided into saponifiable and non-saponifiable lipids. Glycolipids, acylglycerols, fatty acids, sterols, and phospholipids are saponifiable lipids. Tocopherols and carotenoids are non-saponifiable lipids [24,29].

Palatability, including aroma, texture or juiciness, and taste are improved by lipids, since they carry fat-soluble flavor molecules. The satiety value of foods is increased by the slow movement of lipids in the gastrointestinal tract [29,39]. Lipids play an essential role in our diet through vital biochemical and physiological processes. They are a source of high energy and form the structure of cell membranes. Fats, oils, specific fat-soluble vitamins, hormones, and most non-protein membrane components are lipids.

### 2.4. Minerals

Over two billion people suffer from micronutrient deficiency according to the World Health Organisation (WHO). The most predominant micronutrient deficiencies are iron, zinc, iodine, and vitamin A [40]. Proximally a third of the world’s population is affected by iron and zinc deficiencies [41]. The determining factor in mineral malnutrition is insufficient dietary intake, so nutrition is the most potent environmental factor that can be targeted to reduce the problem over the course of an individual’s lifetime [42,43].

Iron is concentrated in the aleurone and zinc in the embryo [44]. Deficiencies in iron and zinc micronutrients are common in populations that consume wheat as a staple because wheat products are usually low in bioavailable forms of these micronutrients. In wheat, two features contribute largely to the low content in bioavailable iron and zinc: the most consumed form is white flour, which contains low concentrations of these minerals, and the existence of phytates in mineral-rich bran fractions that retain minerals in a form that is not bioavailable.

Interest in improving the mineral and vitamin contents of cereal crops has been growing in the last 20 years [45]. Genetic modification and conventional breeding are the two main biofortification strategies. Conventional breeding in conjunction with foliar application of ZnSO_4_ was used to develop high-zinc types of wheat [46]. This approach has not worked for iron. Transgenic approaches have increased iron and zinc contents in white flour by transforming the starchy endosperm tissue into a “sink” for minerals [47]. The overexpression of metal transporter genes increased single mineral content in starchy endosperm cells according to the respective highly specific metal transporters targeted. For example, expression of a wheat vacuolar iron transporter (TaVIT2) under the control of an endosperm-specific promoter more than doubled the iron content of the white flour fraction [48], while expression of the barley metal tolerance protein 1 (HvMTP1) using an identical promoter considerably increased the zinc content in the endosperm of barley grains [49].

Moreover, selenium (Se) is an essential micronutrient for regular cell metabolism in animals and humans, being present as selenocysteine in several enzymes [50], but no function is known in plants. For over a century, Se was known only as a toxin [51], but in the late 1950s, it was first recognized as an essential micronutrient for animals [52]. Many people worldwide have a low dietary intake of Se. This is due to the low bioavailability of Se in some soils and, therefore, low Se concentrations in plant tissues. In livestock, Se deficiency is common, causing diseases such as white-muscle disease in cattle and sheep. In humans, severe Se deficiency has been associated with two conditions: Keshan disease, a cardiomyopathy occurring in people living in a geographic area stretching from north-east to south-west China [53,54,55], and Kaschin-Beck disease, an osteoarthropathy occurring in China and less widely in south-east Siberia [53,56].

In most diets, meats, fish, and cereals are the primary sources of Se [54]. Wheat grain Se concentration can vary from about 3 µg/kg in places like the Keshan disease area in China where the Se concentrations in staple crops and Se availability in soils are very low, to over 2000 µg/kg in North and South Dakota in the USA [54]. The minimum nutritional level for humans and animals is about 50–100 µg Se/kg in dry food, and intake below this range may cause Se deficiency [57].

Biofortification strategies to improve wheat Se content have been employed to diminished Se deficiency and those public-related health issues. Using a meta-analysis approach, Ros et al. [58] showed that fertilizers based on selenate could increase Se uptake by crops and consequently Se intake in humans and animals. In Finland, Se biofortification approaches have been practiced commercially in regions deficient in Se by adding to soils Se-amended inorganic fertilizers [59,60]. A solution containing Se is dosed onto the crops leaf surface, enriching the Se content in agricultural products [61]. Ros et al. [58] estimated that the most efficient fertilizer method to increase crop Se uptake in most arable crops is the selenate fertilization of the foliar. Genetically-modified plants have also been developed to improve the uptake of Se from the soil. Possible genetic targets for strengthening the Se content of wheat are in the acquisition and distribution processes, usually catalyzed by transporters. Overexpressing genes encoding transporters for selenite, selenate, or seleno-amino acids in the plasma membrane of particular cells can improve Se uptake and transport capacity inside the plant [62].

### 2.5. B Vitamins

The B vitamin complex, which at first was thought to be a single compound, comprises eight water-soluble components, which often co-occur in the same foods. They are unequally spread in the wheat kernel and are primarily found in wheat bran and the germ; hence they are present in reduced quantities in refined flours [29]. Cereals are dietary sources of several B vitamins, particularly riboflavin (B2), folates (B9), thiamine (B1), pyridoxine (B6), and niacin (B3) [21]. These molecules play an essential role in metabolism, particularly thiamine in the metabolism of carbohydrates, and riboflavin and pyridoxine in the metabolism of proteins and fats [63]. Consumption of wholemeal products provides 40% of the recommended daily allowance of thiamine, 10% of riboflavin, 22% of niacin, 33% of vitamin B6, and 13% of folate recommended [64]. Niacin is of particular concern as only a proportion of the total present in cereals is bioavailable in a chemically bound form, nicotinic acid [65]. Multiple studies have focused on this issue [66], with some reports of increasing niacin bioavailability by treatment with alkali [67].

Dietary vitamins are required to prevent deficiency disorders [68]. Many of these deficiency diseases, such as beriberi (B1 deficiency), and pellagra (B3 deficiency), are the most common diseases worldwide, and are particularly frequent in developing countries.

### 2.6. Phytochemicals

Two main groups of phytochemicals, derived from different biosynthetic pathways, are present in wheat grain: phenolics and terpenoids [21].

The primary group of phytochemicals in wheat grain are phenolic acids, but numerous other phenolic compounds have been identified, including lignans, alkylresorcinols, and flavonoids [64]. Phenolic compounds are characterized by at least one aromatic ring carrying at least one hydroxyl group. Phenolic acids have vigorous antioxidant activity, and the total phenolic content is correlated with total antioxidant activity [69,70]. The importance of antioxidant properties for human health is widely discussed; however, evidence that phenolic compounds, including ferulic acid, the major phenolic acid in wheat, improve vascular function in humans is increasing [71,72,73].

Cereals are also significant sources of terpenoids in the form of sterols and tocols. Sterols in wheat and other plant materials can be classified according to structural and biosynthetic characteristics into desmethyl sterols, 4α-monomethyl sterols, and 4,4-dimethyl sterols [74,75,76]. Sterols have the capacity to low cholesterol in humans, the health benefits of which are broadly accepted in Europe. Tocols consist of a chromanol ring with a C16 phytol side chain, which can either be saturated (tocopherols, T) or contain three double bonds at carbons 3, 7, and 11 (tocotrienols, T-3). Although the name “vitamin E” is frequently applied to all tocols, they can be distinguished by their biological activity, with α-tocopherol being the most active form [21]. Vitamin E is the most essential lipid-soluble antioxidant in the human body, and together with other antioxidants such as vitamin C they provide an efficient protective network against oxidative stress. α-tocopherol is the most reactive vitamer and is unstable and the first to be broken down [77]. This may reduce its capacity as a long-term antioxidant in food systems, and a combination of tocols is regularly preferred to ensure antioxidant protection. Other studies have shown that tocotrienols might be similar to or even have more potential than tocopherols as antioxidants [78,79].

### 2.7. Wholegrain

Since the 1900s, when Dr. Thomas Allinson promoted Allinson’s bread as a healthier lifestyle, the intake of wholegrain wheat has been promoted for its health benefits [80]. The interest in studying the wholegrain wheat composition has been increasing in order to identify compounds that promote health and better recognize the full potential of wheat in disease avoidance and health [81]. Wholegrain wheat products include various components with recognized or proposed health benefits, including dietary fiber, phenolic acids, carotenoids, flavonoids, sterols, lignans, selenium, magnesium, alkylresorcinols, tocopherols, and B-complex vitamins [7,69,81,82,83,84,85], which are mainly presented in the bran. Consequently, they are either absent or present in lower amounts in white flour, which is almost exclusively derived from endosperm starch cells [7]. While other whole grains may contain similar components, wheat eminence in the diet potentially makes this cereal a more significant contributor to the intake of these compounds [86,87].

Wholegrain-based foods are a valuable source of dietary fiber. About 1 g of dietary fiber is delivered by a simple side of 40 g of white wheat bread, and a similar serving of wholemeal bread would provide 3–4.5 g of dietary fiber [19,88]. This means that the choice of bread alone has a significant effect on dietary fiber intake [19]. However, cereals are mainly consumed as refined products with lower dietary fiber contents, such that today the daily dietary fiber intake is less than the recommended (25–35 g recommended per day) [89].

Wholegrain wheat may protect against the development of diseases related to chronic diet. Extensive cohort studies have described a noticeably reduced risk of cardiovascular disease [15,90,91], type 2 diabetes [14,17,92], and certain forms of cancer [93,94,95] with increased consumption of wholegrains, and wheat has been identified as a critical food in creating these results [96].

## 3. Wheat/Gluten-Related Disorders

**Figure 2:**
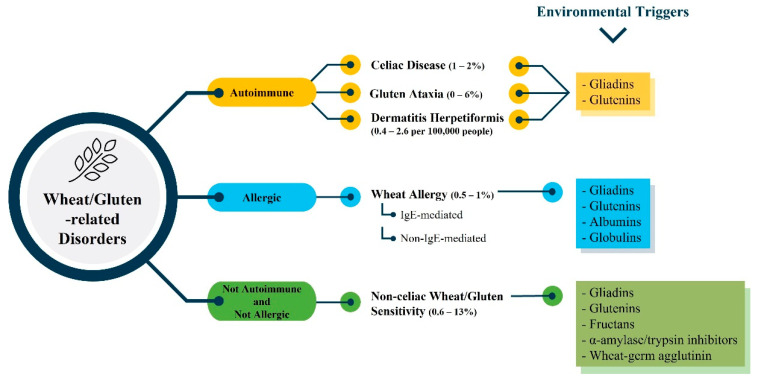
Wheat/gluten-related disorders, their prevalence and environmental triggers.

Wheat/gluten-related diseases can be classified into three different disorders: autoimmune, allergic, and neither autoimmune nor allergic (Figure 2). Celiac disease is the most prominent autoimmune gluten-related disorder (CD). It is a condition of the small intestine caused by gluten and gluten-related proteins and influenced by environmental and genetic factors [97,98]. An IgE and non-IgE mediated immune response characterize wheat allergy (WA), resulting in an allergic reaction in some individuals upon contact, inhalation, or uptake of foods containing wheat but not necessarily other grains as barley or rye. However, IgE-cross reactivity to other cereals is possible in some people [99,100,101]. Patients with non-celiac wheat/gluten sensitivity (NCWGS) experience identical symptoms to CD, but they do not test positive for CD [102].

### 3.1. Celiac Disease

The binding of gluten peptides to T cells triggers CD in some individuals expressing human leukocyte antigen (HLA) DQ2 or DQ8 in cells specialized in presenting antigens. Specific CD4+ T cells then recognize the presented peptides releasing inflammatory cytokines, leading to changes in the architecture of intestinal mucosa with atrophy and flattening of villi that can lead to total villous degeneration and enteropathy. Moreover, gliadin peptides are responsible for the activation of innate immunity of the intestinal epithelial cells [103,104]. Hence, the gliadin peptides can directly stimulate the immune response of macrophages and dendritic cells through pattern recognition receptors (PRR), such as toll-like receptors (TLRs) 4 [105]. It has also been demonstrated that tissue transglutaminase, an enzyme involved in the deamidation of glutamine residues to glutamate, present in the intestinal epithelium, plays an important role by increasing the binding affinity of gluten peptides to HLA-DQ2 and DQ8 heterodimeric receptors [7].

The expression of the major histocompatibility complex (MHC) class II molecules is related to genetic risk factors. HLA-DQ2 and HLA-DQ8 are the most potent genetic risk factor in CD since they are critical in initiating detrimental immune responses [98]. Nevertheless, additional genetic variations are reported as risk factors in CD, which means HLA-DQ2 and HLA-DQ8 do not account for all the genetic susceptibility to CD [106]. For example, two cytokines implicated in CD pathogenesis are encoded by specific gene polymorphisms of IL-2/IL-21 [107]. In the past, non-HLA genetic risk factors were reported [108]. The consumption of gluten and gluten-related proteins is the main environmental risk factor for CD, which employs a strong immunodominant function in people with a genetic susceptibility to CD [98]. Studies of the timing of gluten introduction in infants’ diets suggest that infants who began to receive gluten either before four months or after seven months of age have more risk of developing CD. This conclusion supports the notion that there is a time-space between four and seven months of age during which the introduction of gluten might induce the tolerance to CD [109,110,111]. However, more recent studies refute this statement, showing no evidence that avoidance of either early (at four months of age) or late (at/after six or even twelve months) gluten introduction puts children at risk of CD [112,113,114,115]. Environmental factors can also play a pathogenetic role in the disease. Studies conducted on twins showed that, in 25% of the cases, one of the two twins did not develop CD, supporting this environmental hypothesis [116].

It is well known that the gut microbiota, which is the microorganisms colonizing the human gut, contribute to the development and function of the immune system, being vital for the development of adequate protective immune responses against harmful agents and tolerance to harmless antigens [117]. Various studies already associated gut microbiota imbalances with immune homeostasis disruption and the risk of developing immune-mediated diseases, such as celiac disease (CD), among others [118]. In addition, modifications of the intestinal microbiota during pediatric age [119,120], neonatal infections [121], and recurrent infections involving rotavirus [122,123] were associated with an increased prevalence of CD. A recent longitudinal prospective cohort study developed by Leonard et al. have analysed the gut microbiota of infants at risk of CD to track shifts in the microbiota before CD development. Comparing 10 infants who developed CD and 10 infants who did not, the researchers identified complex patterns of increased abundances of proinflammatory species and decreased abundances of protective and anti-inflammatory species at various time points preceding the onset of the disease. They believe these microbiome shifts, coupled with metabolome findings, may represent potential biomarkers of CD development [124]. However, further studies are necessary since the number of patients examined was low.

#### 3.1.1. Diagnosis

The most common symptoms in adults and children with CD are diarrhea, which may be persistent or intermittent, abdominal pain, fatigue, abdominal distension, vomiting and nausea, constipation, bloating and gas, and weight loss [125]. Gain of gluten intolerance can arise at any time in life due to additional triggers apart from gluten. Trigger factors such as α-interferon, gastrointestinal infections, medications, and surgery have all been indicated [126,127,128]. For CD diagnosis, a combination of clinical, serological tests and duodenal biopsies is required to check for damage in the intestine caused by the disease [98,129]. Patients with a clinical demonstration of CD should be submitted to serological tests [11,130]. The recommended serological test for the detection of CD is IgA-tTG. As IgA-deficiency affects 2–3% of CD patients and leads to false-negative results, total IgA levels also need to be measured [131,132]. Other antibodies can be used such as anti-deamidated gliadin (anti-DGP), anti-tissue transglutaminase (tTG), and anti-endomysial (EM) antibodies [115,133,134,135]. In the presence of IgA-deficiency, IgG antibody-based tests (IgG-tTG and/or IgG-DGP) should be used [132]. Depending on the antibody test a duodenal biopsy is performed to establish the definitive diagnosis of CD [136,137]. In agreement with the Marsh classification villous atrophy, hyperplasia, and increased intraepithelial lymphocytes on the duodenal biopsy together to positive serological results confirm celiac disease diagnosis [138]. HLA typing can be used when the results of the serological and duodenal biopsies are inconclusive and the diagnosis of CD is uncertain [139,140].The determination of HLA forms is effective along with histological findings [141,142], knowing that HLA-DQ2 and HLA-DQ8 molecules are correlated with ~95% and 5% CD patients, respectively [142,143].

#### 3.1.2. Treatment

The only currently available therapy for CD is adherence to a gluten-free diet (GFD). A GFD consists of complete avoidance of gluten and gluten-related proteins, which rules out wheat, rye, barley, and any products containing them [131]. Proper adherence to a GFD usually improves the clinical symptoms, increases bone density, and improves body weight distribution or nutritional status [144]. After long-term adherence to GFD, the intestinal villi can be significantly reconstituted [145]. However, GFD may be associated with digestive problems such as constipation due to a low fiber intake. In many cases, a strict GFD is onerous to follow due to gluten residues in certain food products [146,147].

Alternative treatments for CD are being development and the most promising strategies include mechanisms to improve the intestine’s permeability, detoxify gluten, or induce modifications in the immune response to gluten [3,98].

### 3.2. Other Autoimmune Wheat/Gluten-Related Diseases

#### 3.2.1. Gluten Ataxia

Gluten ataxia (GA) is a form of cerebellar ataxia, affecting mainly Purkinje cells, and is caused by antibodies released when digesting gluten that mistakenly attacks part of the brain in individuals that are sensitive and genetically susceptible [148]. The clinical symptoms of GA are identical to those of other ataxias. They include gait ataxia (100%), lower limb ataxia (90%), gaze-evoked nystagmus (84%), upper limb ataxia (75%), ocular signs like dysarthria (66%), and other movement disorders including chorea, myoclonus, opsoclonus myoclonus, and palatal tremor [149].

##### Diagnosis

GA diagnosis is supported when anti-tTG, anti-gliadin, and anti-TG6 (anti-transglutaminase 6) antibodies are found in the serum. The best diagnostic approach for patients with suspected GA remains unclear. Still, one study reported that considering the whole spectrum of gluten, the IgG anti-gliadin antibody performs better than the gluten ataxia marker because of its elevated sensitivity [149].

Studies of GA patients have shown that the brain presents anti-tTG antibodies. If CD serology is positive, it is necessary to look for evidence of CD through an intestinal biopsy [150]. Magnetic resonance imaging (MRI) can be utilized to diagnose GA. MRI results from up to 60% of GA patients show evidence of moderate cerebellar atrophy [151].

##### Treatment

Following a rigorous GFD should be the treatment to GA patients. Moreover, studies show that immunotherapy with steroid and intravenous immunoglobulins (IVIG) can be an efficient treatment for such patients [152].

#### 3.2.2. Dermatitis Herpetiformis

Dermatitis herpetiformis (DH), repeatedly associated with CD, is an autoimmune, chronic, and recurrent cutaneous-intestinal disorder detected in genetically susceptible individuals [11,153,154]. Anti-tTG antibodies that also recognize epidermal transglutaminase (ETG) can be produced after exposure to gluten. ETG is homologous to tTG in terms of structure and is the primary antigen in DH [153]. IgA antibody deposition in dermal papillae causes pruritic, vesiculobullous, and localized lesions in DH patients. DH affects the extensor surfaces such as knees, buttocks, elbows, and scapular areas [153,155,156].

##### Diagnosis

Patients with clinical symptoms are advised to undergo direct immunofluorescence (DIF) tests on perilesional skin. If the test result is negative, new material is collected, and it is determined whether the patient is on a GFD, which could lead to false-negative results [157]. Other confirmatory tests such as the dosage of anti-tTG can be used in patients with symptoms suggesting DH but with negative direct immunofluorescence [158].

##### Treatment

Like CD patients, DH patients have the same HLA haplotypes (DQ2 and DQ8) and following a GFD improves the symptoms [154]. Drug therapy with dapsone or sulfonamides is also a possible treatment [153].

### 3.3. Wheat Allergy

Allergens cause allergic reactions, and wheat is one of the five most frequent foods causing them in children. After milk and eggs, wheat is the most common allergen in Japan, Germany, and Finland [159]. In children and adults, wheat allergy (WA) prevalence is approximately 1% depending on age and region [160,161]. In contrast to CD, distinct wheat components such as water-insoluble proteins (gliadin and glutenin) and water/saline-soluble proteins (albumin and globulin) contribute to the development of WA [11,162,163].

#### 3.3.1. IgE-Mediated Wheat Allergy

IgE-mediated WA is triggered by allergen ingestion (food allergy), inhalation (respiratory allergy), or skin contact (dermal allergy). The antigen is introduced by dendritic cells that trigger CD4+ T cells to differentiate into T helper type 2 (Th2) cells. These cells produce cytokines such as IL-4, IL-5, and IL-13 that stimulate B cells to produce IgE [142,164]. When a new exposure to wheat allergens occurs, the IgE antibodies that are bound to their high-affinity receptor (FcԑRI) on basophils or mast cells, recognize specific epitopes in wheat allergens [165]. The recognition results in IgE-crosslinking that triggers the release of vasoactive mediators like histamine from mast cells or basophils, leading to allergic responses, including WA [166,167]. The most common symptoms of WA due to these mechanisms include gastrointestinal symptoms (nausea, abdominal pain, vomiting, diarrhea), dermal (itching, eczema, redness), respiratory (rhinitis, asthma), circulatory (flushing, angioedema), and cerebral (disturbed thinking, headache, dizziness) which typically manifest minutes to hours after exposure [164,168].

Wheat-dependent exercise-induced anaphylaxis (WDEIA) is a particular type of IgE-mediated WA. This condition gives rise to severe anaphylactic reactions to wheat when intense exercise is practiced soon after being consumed [169]. Symptoms of WDEIA include angioedema, chest pain, diarrhea, dysphagia, dyspnea, flushing, headache, hoarseness, nausea, pruritus, and syncope [142,170].

Baker’s asthma is also an IgE-mediated WA that develops after allergen inhalation, especially cereal flour dust present in the work environment, and affects 0.03–0.24% of pastry factory workers, cereal handlers, confectioners, and bakery workers. It is considered one of the most frequent occupational, cereal-induced allergic asthmas [171,172,173]. Consuming cooked wheat or products containing it does not manifest symptoms in these patients, but they may react after eating products contaminated with raw wheat flour [174].

IgE-mediated wheat allergens are widely distributed in wheat’s different protein fractions. Currently, 28 allergens have been identified in wheat, according to WHO/IUIS Allergen Nomenclature Sub-Committee (Table 2).

The heat-resistant α-amylase/trypsin inhibitor is an allergen that binds to specific IgE and is involved in anaphylaxis, in some cases of WDEIA [175], and baker’s asthma [176]. Wheat seeds highly express Tri a 37, which is a plant defence protein. It is also resistant to digestion and heat and can act as a powerful allergen. Individuals who have IgE antibodies against Tri a 37 have a high risk of severe allergic symptoms upon wheat intake [177,178]. ω-5-gliadin, also known as Tri a 19, is involved in anaphylactic reactions to wheat and WDEIA in children [179,180].

##### Diagnosis

The diagnosis of WA in its various clinical presentations (whether allergy associated with wheat ingestion, baker’s asthma, or WDEIA) depend on taking a detailed clinical history, physical examination, and selecting the proper tests.

The first examinations include a skin prick test and measurement of specific IgE to wheat allergens in wheat extracts and blood serum. In case of wheat allergy due to ingestion, the results of the tests and the clinical history may prompt an oral food challenge. The double-blind placebo-controlled wheat challenge continues to be the gold standard. Oral food challenges are generally considered secure, but experts must perform them prudently because anaphylactic reactions may happen [181]. For diagnosis of WDEIA, in addition to establishing an accurate clinical history, tests for specific IgE against wheat and specific wheat allergens, such as ω-5-gliadin, are performed. These patients may also need to complete a placebo-controlled wheat/exercise challenge, which involves the controlled ingestion of wheat followed 30 min later by 15–20 min of exercise on the treadmill [168,170]. The standard gold diagnosis of baker’s asthma is the bronchial challenge test in which patients test positive. In addition, experts establish the clinical history followed by the confirmation of specific IgE to wheat in serum and/or by skin prick test [168].

##### Treatment

Complete elimination of wheat from the diet is the only available therapy to treat IgE-mediated WA. In allergy associated with wheat ingestion, patients should follow an adequate wheat elimination diet and be trained in the correct interpretation of product labels [168]. To prevent WDEIA, patients must avoid wheat consumption in any circumstance, but if not, they can only exercise 6 h after the consumption of wheat or wheat-containing products [170]. In the case of baker’s asthma, a total restriction of exposure to wheat flours is recommended [168].

However, in many cases, strict avoidance of wheat is challenging because wheat is present in so many distinct food products, and involuntary exposure to small traces can occur. Currently, new approaches to treat IgE-mediated WA are actively being sought. Immunotherapy is a promising treatment based on the administration of increasing amounts of an allergenic source to regulate the immune system and achieve remission of allergic symptoms [182]. Three distinct types of immunotherapy are currently being tested: sublingual immunotherapy (SLIT), oral immunotherapy (OIT), and epicutaneous immunotherapy (EPIT). In SLIT and OIT the amount of food ingested is gradually increased to avoid the induction of systemic reactions, while EPIT involves delivering the allergen to the patient using a skin patch [183].

#### 3.3.2. Non-IgE-Mediated Wheat Allergy

Non-IgE-mediated wheat allergy usually occurs 2 h after ingestion of wheat. It is strongly associated with eosinophilic esophagitis (EoE) or eosinophilic gastritis (EG), which occur when eosinophils infiltrate the gastrointestinal tract [168]. Typical manifestations of this type of WA are indigestion, diarrhea, vomiting, arthralgia, and headaches that can appear numerous hours or days after consumption of allergens [11].

##### Diagnosis

To diagnose EoE, when suspected, an esophageal biopsy is performed by an esophagogastroduodenoscopy (EGD), and it is necessary to find 15 eosinophils per high-power field (eos/hpf) at least. However, the identification of which food causes EoE is more difficult. The gold standard remains: an EGD performance eight weeks after an elimination diet to assess the significance of a food allergen in EoE pathogenesis [168,184,185]. The EG diagnosis is made when clinical symptoms suggest it. Then to confirm a positive diagnosis, a biopsy must show eosinophilic inflammation whit 30 eos/hpf in the stomach and 50 eos/hpf in the duodenum [168,186].

##### Treatment

The currently accepted treatment to EoE is similar to other atopic diseases and is based on corticosteroid use and allergen avoidance. To treat EoE, steroid treatment for an IgE-mediated food allergy is one convenient approach. Three accepted nutritional strategies can also be used to treat this disease: (1) an elemental diet in which only essential formulas are ingested; (2) avoidance of specific antigens according to allergy testing results and/or diet history; and (3) empiric food elimination of the most common food antigens [187,188]. The adaptation of an EG diet through empiric dietary elimination therapy, consisting of exclusion of common food triggers established for EoE, and very restrictive therapies, consisting of amino acid-based formula ingestion with a few foods, has been tried by pediatric and adult patients, and found to be effective in the majority of pediatric patients [189]. Nevertheless, diet alone is infrequently an effective therapy due to the severity of the symptoms and steroids are necessary to rapidly reduce them. For this reason, the majority of patients are primarily treated with systemic steroids (0.5–1 mg/kg/day for 5–14 days) followed by a gradual decrease over 2–4 weeks [168].

### 3.4. Non-Celiac Wheat/Gluten Sensitivity

Non-celiac wheat/gluten sensitivity (NCWGS) makes people experience symptoms similar to CD and WA. However, patients with NCWGS do not have specific IgE against wheat proteins or IgA anti-TG2 autoantibodies. The symptoms develop in a few hours or days after wheat/gluten consumption and include abdominal distension, abdominal pain, diarrhea, gas, among others. Patients also experience extraintestinal symptoms, including headache, fatigue, pain in muscles and joints, and eczema [190]. Recent studies have given rise to the idea that other wheat components, such as oligosaccharides like fructans [191], α-amylase/trypsin inhibitors [192], and wheat-germ agglutinin [193], may contribute to the development of NCWGS.

The pathogenic mechanisms of NCWGS are far from understood. Preliminary data indicate that activation of innate immunity triggers NCWGS without the involvement of adaptive immunity, which would be a crucial factor in CD development [194,195,196]. The increased expression of toll-like-receptors (TLRs), a protein class that plays a vital role in innate immunity, in the small intestine is the evidence supporting the hypothesis of the activation of innate immunity in NCWGS. TLR2, TLR1, and TLR4 have been identified in the intestinal mucosa and some cells of the lamina propria of patients with NCWGS [194]. There is diverging information on intestinal permeability in NCWGS. A study conducted in 2011 determined the gut permeability of NCWGS and CD patients using the urine lactulose/mannitol test. The small intestines of NCWGS patients were significantly less permeable than those of CD patients and controls. Moreover, duodenal biopsies of NCWGS patients found higher expression of claudin-4 mRNA, a marker of reduced permeability [194]. By comparison, another study reported a subgroup of HLA-DQ2/DQ8+ patients with diarrhea-predominant irritable bowel syndrome following a gluten challenge that had increased intestinal permeability [197]. Moreover, Hollon et al. (2015), in an ex vivo study, evaluated alterations in transepithelial electrical resistance (TEER) of tissue biopsies from NCWGS patients, active CD patients, CD patients in remission and controls subjected to pepsin-trypsin digested gliadin. This study has shown that exposure to gliadin increases intestinal permeability and decreases TEER in all patient groups compared to controls [198]. This discrepancy suggests that further studies are required to define the small intestine’s permeability in NCWGS and improve our overall knowledge about it.

#### 3.4.1. Diagnosis

Currently, the lack of diagnostic biomarkers for NCWGS means that diagnosis depends on a clinical symptoms evaluation and elimination of CD and WA. According to the Salerno Experts’ criteria, first, patients have to adhere to a wheat/gluten exclusion or wheat/gluten-reduced diet in order to reduce the symptoms. Then, to confirm the diagnosis, a double-blind, placebo-controlled gluten challenge must be performed to determine if symptoms were indeed related to wheat/gluten ingestion [199,200]. About half of patients with NCWGS present the first generation antibody to gliadin (AGA) which is considered the only serological marker [199,201,202]. Nevertheless, testing for the presence of AGA is not a specific analysis to diagnose NCWGS. Still, for the moment, its positivity, particularly at a high titer, in suspected NCWGS patients can support the diagnosis [203].

Not long ago, Kabbani et al. reported a diagnostic algorithm based on the specific combination of the presence or absence of several histological, serological, and clinical markers to identify NCWGS and distinguish it from CD. The authors concluded that patients with negative celiac serologies (no IgA/IgG deaminated gliadin peptide or IgA tTG antibody) on a regular diet are improbable to have CD. Those with negative serology who also do not have a clinical indication of malabsorption and CD risk factors are likely to have NCWGS and may not necessitate additional examination. Those with ambiguous serology should be subjected to an HLA typing to establish the requirement for biopsy [204].

A recent discovery has given hope to future NCWGS diagnoses. This study developed by Barbaro et al. verified that NCWGS and CD patients had significantly increased levels of zonulin compared with asymptomatic controls and diarrhea-predominant irritable bowel syndrome (IBS-D) patients. They came to the conclusion that zonulin can be considered a diagnostic biomarker in NCWGS and combined with demographic and clinical data, differentiates NCWGS from IBS-D with high efficiency. Moreover, wheat withdrawal was associated with reducing zonulin levels only in NCWGS carrying HLA genotype [205]. However, further studies are necessary since the number of patients examined was low.

#### 3.4.2. Treatment

The guidelines to treat NCWGS patients are not established yet. The specialists advise these patients to adjust their dietary preferences and begin a GFD [203]. In some cases, any progress after GFD is only partial. In these situations, a low FODMAP (fermentable oligosaccharides, disaccharides, monosaccharides, and polyols) diet together with gluten removal can enhance the clinical condition considerably [206].

Significant research efforts are being made to manage NCWGS. For example, multiple studies have concentrated on analyzing the toxicity of different varieties of wheat. Intriguingly, Triticum monococcum ssp. monococcum, an ancient diploid wheat, does not activate distinct immune cells involved in gluten-related disorders as much [207]. While clinical studies have shown that CD patients cannot consume these varieties, it has been indicated that they would be safe for patients with NCWGS [208]. Innovative hybridized cereals such as tritordeum have also been shown to be an alternative for NCWGS patients due to their low gliadin content [209]. CRISPR/Cas9 technology has been used to produce wheat with less α-gliadin, translating into an 85% reduction in immunoreactivity [210]. Nevertheless, it should be mentioned that most of this research is designed to tackle gluten proteins, particularly gliadins, and their post-ingestion downstream effects in CD. In NCWGS, the environmental culprit is yet to be well defined.

## 4. Gluten-Related Misconceptions

GFDs are commonly recognized as the treatment for CD and other gluten-related disorders (GRD), as mentioned above. However, nowadays, the number of people without any GRD who adopt a GFD is rising [211]. The prevalence of adherence to a GFD in the overall adult population can reach 7% in a few countries [212,213]. As of 7th December 2020, a Google search for “gluten-free diet” generated over 4.5 million results. The general population’s principal reason for purchasing gluten-free foods is that they are supposed to be healthier than their gluten-containing equivalents [214]. Recommendations from a multitude of books, celebrities, and other media have unquestionably supported the increased consciousness of the potential health benefits of gluten avoidance, such as weight loss [215].

There are three significant misconceptions by the general population leading them to follow a GFD. (1) A gluten-free diet is a healthier option. (2) Eating gluten-free will help them lose weight. (3) The wheat we consume today contains more gluten than older varieties.

Considering the first misconception, claims of the potential benefits of following a GFD include increased energy, better sleep, clearer skin, faster weight loss, and improved medical conditions such as autism and rheumatoid arthritis [214]. Evidence of the health benefits of a GFD for GRD patients is incontrovertible. However, no published experimental evidence supports similar claims for the overall population [216]. On the contrary, an issue associated with unnecessary gluten avoidance is the reduced consumption of whole grains, foregoing the likely benefit of lowering cardiovascular risk. The GFD promotion between people without CD must not be encouraged [217].

Regarding the second misconception, some studies of CD patients report a change in weight as an effect of following a GFD. In a survey of 369 adult patients with CD who followed a GFD for an average of 2.8 years, 22 of the 81 (27%) who were at first overweight increased weight [218]. In another study of 371 adults with CD who adhere to a GFD for two years, 55 of the 67 (82%) initially overweight patients earned weight [219]. Other researchers have reported that between 149 children with CD adhering to a GFD for at least 12 months, the percentage of overweight people almost duplicated (11% to 21%) [220]. These studies suggest that body weight may increase for a considerable portion of overweight celiac patients while on a GFD. However, it has not been established if people without CD or gluten sensitivity would gain weight if they followed a GFD. In this respect, it is essential to note that gluten-free does not imply fat-free or calorie-free, and some gluten-free products contain more calories and sugar than corresponding gluten-containing foods [214]. Indeed, a study carried out in 2018 analyzed the most recent surveys on the nutritional quality of gluten-free products and concluded that the key inadequacies of currently available GF products are a low protein content and a high fat and salt content compared with their gluten-containing counterparts. However, they also verified more acceptable levels of fiber and sugar than in the past [221]. In this way, we can affirm that gluten-free products are not adequated to people wishing to lose weight.

The third misconception that wheat breeding has led to the production of wheat varieties containing higher levels of gluten originated from successful books like “Wheat Belly” by William Davis and “Grain Brain” by David Perlmutter [222]. However, the level of gluten in wheat has actually remained unchanged over the years. A 2013 study reported that gluten levels in numerous varieties, on average, have slightly changed since the 1920s, and although there was actually an increase in CD in the second half of the century, the breeding of wheat for higher gluten content does not suggest to be the reason for that [223]. In 2010, van den Broeck, when studying old and modern wheat varieties toxicity, suggested that breeding practices may have influenced the increased CD prevalence. However, some evidence has shown that modern wheat is not more toxic for celiac patients and that breeding does not seem to be related to a higher prevalence of CD [224]. On the other hand, as nitrogen (N) fertilization of cereal crops has increased, another hypothesis has emerged. Intensified fertilization with N may increase the allergenic proteins content of wheat, which may be related to the increase in CD pathology. The study that put forward this hypothesis concluded, after a literature meta-analysis, that wheat grown under higher N availability in the soil produces not only higher yield but also grains and flour with higher concentrations of gliadin in all genotypes [225]. However, further experimental studies need to be done, and if this hypothesis stands, we will have an important lead to follow to prevent and control the spread of CD.

## 5. Conclusions

Wheat is the widest cultivated crop on Earth and has been consumed for 10,000 years by humans from its most primitive form to the current species. Wheat is a nutritious cereal, rich in dietary fiber. The nutritional recommendations of many countries emphasize cereals as the basis of a balanced diet. This is particularly true in low and medium-income countries where grain-based food is the main source of energy, carbohydrates, fibers, proteins, B vitamins, and minerals essential for human survival. The exclusive properties of dough made from wheat flour derive from the gluten protein complex and allow it to be processed into bread, pasta and noodles, and other diverse forms of food feeding most of the world population.

Considering the predominance of wheat, the challenge of the increasing incidence of wheat/gluten-related disorders like CD and NCWGS must be addressed now. Patients with CD should strictly follow GFD since they must avoid foods containing gluten, patients with a WA should prevent contact with any form of wheat, and NCWGS patients should follow a wheat/gluten exclusion diet as well. In some cases, adherence to a low FODMAP diet and gluten removal can drastically improve the clinical outlook. There have been many research advances in improving CD and WA diagnosis, but the same does not happen at NCWGS. Thus, first, we have to comprehend the fundamental mechanism behind the NCWGS pathogenicity to establish more sensitive diagnostic markers and therapeutics then.

Only a tiny percentage of the worldwide population is affected by these wheat/gluten-related disorders. Opting or promoting a GFD to improve well-being unwarranted by any medical suggestion is an unhealthy alternative since the consumption of wheat is more beneficial than its non-consumption. Thus, to answer the question “How healthy is to eat wheat?” and the take-home message is that wheat is an excellent food for people without any associated medical conditions because it is a very nutritious cereal, rich in macro and micronutrients that only beneficiate our health. The problem is that people are removing wheat from the diet without any medical indication or health/nutritional condition with a proven relationship and consequently are not consuming the necessary nutrients. This is a mistake that results from a growing number of misconceptions related to this cereal that should be avoided and clarified as they end up harming these people’s health. Here we have presented the medical conditions and the nutritional benefits of consuming wheat, so readers can access unbiased information that clearly shows the best and the worst of this cereal in terms of nutrition and health.

## Figures and Tables

**Table 1:** Essential amino acid levels recommended for older children, adolescents, and adults compared with those in whole wheat, wholemeal wheat, and white flour (expressed as g/g protein).

Amino Acids	FAO Recommended Intake Levels			Amino Acids Content		
	Older Children (Age 11–14)	Adolescents (Age 15–18)	Adults (Age > 18)	Whole Wheat	Wholemeal Wheat	White Flour
Histidine	0.016	0.016	0.015	0.022	0.0266	0.0269
Isoleucine	0.030	0.030	0.030	0.038	0.0314	0.0309
Leucine	0.061	0.060	0.059	0.067	0.0594	0.0565
Lysine	0.048	0.047	0.045	0.027	0.0288	0.0222
Methionine + cysteine (sulfur amino acids)	0.023	0.023	0.022	0.039	0.0363	0.033
Phenylalanine + tyrosine (aromatic amino acids)	0.041	0.040	0.038	0.077	0.0544	0.0514
Threonine	0.025	0.024	0.023	0.029	0.0254	0.0224
Tryptophan	0.0066	0.0063	0.006	0.012	-	0.0085
Valine	0.040	0.040	0.039	0.047	0.0388	0.0354
Authors (Reference)	Food and Agriculture Organization of the United Nations [27]			Khan et al. [4]	Shewry et al. [21]	

**Table 2:** Wheat proteins implicated in IgE-mediated wheat allergy. Source: www.allergen.org (accessed on 19 July 2021).

Wheat Allergen	Biochemical Name	Molecular Weight (kDa)	Route of Allergen Exposure
Tri a 12	Profilin	14	Food
Tri a 14	Non-specific lipid transfer protein 1	9	Food
Tri a 15	Monomeric alpha-amylase inhibitor 0.28		Airway
Tri a 17	Beta-amylase 56	56	Food
Tri a 18	Agglutinin isolectin 1		Food
Tri a 19	Omega-5 gliadin, seed storage protein	65	Food
Tri a 20	Gamma gliadin	35 to 38	Food
Tri a 21	Alpha-beta-gliadin		Food
Tri a 25	Thioredoxin		Food
Tri a 26	High molecular weight glutenin	88	Food
Tri a 27	Thiol reductase homolog	27	Food
Tri a 28	Dimeric alpha-amylase inhibitor 0.19	13	Food
Tri a 29	Tetrameric alpha-amylase inhibitor CM1/CM2	13	Airway
Tri a 30	Tetrameric alpha-amylase inhibitor CM3	16	Airway
Tri a 31	Triosephosphate isomerase		Airway
Tri a 32	1-Cys-peroxiredoxin		Airway
Tri a 33	Serpin		Airway
Tri a 34	Glyceraldehyde-3-phosphate dehydrogenase		Airway
Tri a 35	Dehydrin		Airway
Tri a 36	Low molecular weight glutenin GluB3-23	40	Food
Tri a 37	Alpha purothionin	12	Food
Tri a 39	Serine protease inhibitor-like protein		Airway
Tri a 40	Chloroform/methanol-soluble (CM) 17 protein [alpha-amylase inhibitor]	15.96	Airway
Tri a 41	Mitochondrial ubiquitin ligase activator of NFKB 1		Food
Tri a 42	Hypothetical protein from cDNA		Food
Tri a 43	Hypothetical protein from cDNA		Food
Tri a 44	Endosperm transfer cell specific PR60 precursor		Food
Tri a 45	Elongation factor 1 (EIF1)		Food
